# Biological determinants of blood-based biomarker levels in Alzheimer’s disease: role of nutrition, inflammation, and metabolic factors

**DOI:** 10.3389/fnagi.2025.1614962

**Published:** 2025-11-06

**Authors:** Aparna Inamdar, Parashuram Bugadannavar, Mahesh Palled, Savita Umarani, Preeti Salve, Bannimath Gurupadayya, Priyanka Patil, Himanshu Sharma

**Affiliations:** 1 Department of Pharmaceutical Chemistry, JSS College of Pharmacy, Mysuru, JSS Academy of Higher Education and Research, Mysuru, India; 2 Department of Pharmaceutical Chemistry, KLE College of Pharmacy, KLE Academy of Higher Education and Research, Belagavi, India; 3 Department of Pharmacy Practice, Manipal College of Pharmaceutical Sciences, Manipal Academy of Higher Education, Manipal, India; 4 Department of Pharmacology, KLE College of Pharmacy, KLE Academy of Higher Education and Research, Belagavi, India; 5 Department of Pharmacology, Teerthanker Mahaveer College of Pharmacy, Teerthanker Mahaveer University, Moradabad, India; 6 Department of Pharmacology, Nims Institute of Pharmacy, Nims University Rajasthan, Jaipur, India

**Keywords:** Alzheimer’s disease, blood-based biomarkers, nutritional factors, inflammation, metabolism, biomarker variability, personalized medicine

## Abstract

**Objectives:**

The review discusses the effect of biological determinants such as nutritional deficiency, systemic inflammation, and metabolic disorders affect blood-based biomarker (BBBM) levels, influencing their use in diagnosing, prognosticating, and treatment in Alzheimer’s disease (AD). While the individual contributions of neuroinflammation, brain insulin resistance, and micronutrient deficiencies to AD pathology are well-established, a significant knowledge gap exists in understanding their intricate, synergistic interactions. This review proposes a novel integrated framework of bidirectional crosstalk where these three factors create a self-perpetuating cycle of neurodegeneration.

**Methods:**

A comprehensive literature review was conducted, including all aspects of epidemiological and biological context associated with vitamins, micronutrients, and dietary patterns; inflammatory cytokines; insulin resistance; metabolic syndrome; and hormonal changes. Emerging integrative approaches such as multi-omics, AI modeling, and systems biology were also reviewed for their possible refinement in biomarker interpretation.

**Results:**

The results prove that the deprivation of vitamins E, D, B12, and antioxidants contributes to oxidative stress and subsequent neuroinflammation that changes levels of blood-based biomarkers. A chronic state of inflammation caused by cytokines like IL-6, IL-18, and TNF-α represents a major link to the formation of increased amyloid plaques and tau tangles. Metabolically deregulated states, such as insulin resistance, dyslipidemia, and thyroid imbalance, further alter variability in biomarkers. All these factors would act together to affect the expression of key biomarkers-Aβ, p-tau, and neurofilament light chain (NFL). Individualized interpretation, stratified clinical trials, and digital monitoring tools are potentially effective for achieving better diagnostic precision and boosting treatment efficacy.

**Conclusion:**

To a large extent, factors must all be understood thoroughly from multiple biological angles to improve early diagnosis, risk prevention, and treatment personalization in AD. Future studies should develop integrative models that consider nutrition, metabolism, and inflammation to address and fully exploit biomarker utility as well as support precision medicine approaches.

## Graphical abstract

**Figure:**
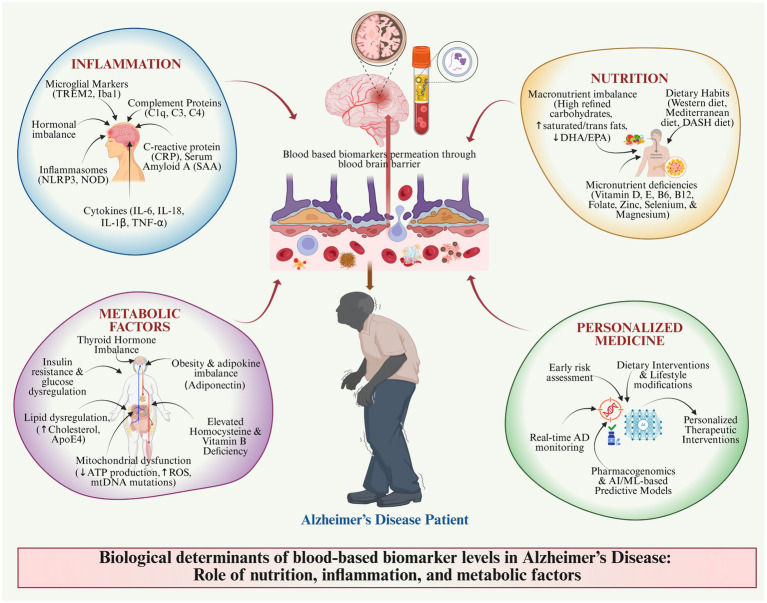
Blood-based biomarker levels and their importance in Alzheimer’s disease.

## 1 Introduction

Alzheimer’s disease (AD) is a type of dementia that affects the brain gradually and hinders appropriate thought processing, resulting in severe memory impairment and physical disability. As people continue to live longer, the incidence of AD is predicted to increase significantly, and therefore, increasingly efficient diagnostic and therapeutic approaches are required. Such blood-based biomarkers have appeared valuable in this case, as they would allow for non-pharmacological detection and surveillance of AD (Inamdar et al., 2025a). This introduction will discuss blood-based biomarkers in AD and their confounders and limitations that, in practice, demand knowledge about the underlying biological factors affecting these biomarkers (Zetterberg and Burnham, 2019).

A biomarker is a measurable biological indicator used primarily to detect diseases. The term “biomarker” was first introduced in 1989, referring to specific biological substances, such as proteins or molecules, whose concentrations can be measured in individuals suspected of having a particular condition. Elevated levels of certain biomarkers in the bloodstream or other bodily fluids indicate a disease process (Alpert, 2011). In the context of AD, the clinical utility of blood-based biomarkers (BBBM) is often limited by their high biological variability. This variability arises from both fixed factors (age, sex, APOE-ε4 genotype) and modifiable influences (nutrition, inflammation, metabolic health), which can shift biomarker concentrations even in the absence of disease progression. For insight, plasma p-tau181 and Aβ42/40 ratios may differ by up to 20–30% between individuals with similar disease burden but different inflammatory or metabolic profiles (Teunissen et al., 2022). Understanding and accounting for such variability is critical to setting diagnostic cut-offs, interpreting longitudinal changes, and avoiding misclassification. Identifying these biomarkers is achieved through a systematic process involving blood sample collection, processing, and laboratory analysis. Of specific biomarkers utilizing techniques such as ELISA for proteins, PCR for DNA or RNA, and mass spectrometry for small molecules and metabolites(Thambisetty and Lovestone, 2010). These methods facilitate the detection and quantification of biomarkers, which are then evaluated against standard ranges. Deviations from normal levels may suggest the presence or progression of AD disease, underscoring the utility of BBBM as a significant tool in diagnosis and management (Mayeux, 2004). The major key determinants like blood-based biomarkers (BBBM), inflammatory markers, systemic inflammation, neuroinflammation, and their definitions are explained in Table 1.

**Table 1:** Key terms and definitions.

Term	Definition	Examples	References
Blood-based biomarkers (BBBM)	Measurable molecules in peripheral blood reflecting pathophysiological processes in the central nervous system, leading to progression of relevant to AD.	Plasma Aβ42/40 ratio, phosphorylated tau (p-tau217), neurofilament light chain (NfL), glial fibrillary acidic protein (GFAP)	Grande et al. (2025); Padala and Newhouse (2023)
Inflammatory markers	Biomarkers reflecting activation of innate or adaptive immune responses may be systemic or specific to the CNS.	Cytokines (IL-6, TNF-α), C-reactive protein (CRP), YKL-40, GFAP	Erichsen et al. (2025)
Systemic inflammation	Evidence of immune activation originating from peripheral compartments, typically measured in blood.	Blood cytokines (IL-6, TNF-α), CRP, and peripheral immune cell activation	Clària et al. (2023)
Neuroinflammation	CNS-restricted inflammatory processes involving astrocytes, microglia, and other glial cells; measurable via CSF or peripheral blood proxies.	CSF or plasma GFAP, YKL-40, sTREM2, microglial activation markers	Roveta et al. (2024)

### 1.1 Importance of blood-based biomarkers (BBBM) in AD

#### 1.1.1 Non-invasive diagnostic tools

The current standard practices for diagnosing AD include neuroimaging (e.g., PET scans) and cerebrospinal fluid (CSF) analysis, which are expensive and inaccessible in many settings. The determination of BBBM is less invasive, making it more patient-friendly. The benefits are proposed in the idea of possible early detection of disease in individuals and consequent treatment, which would change the course of the disease (Hansson et al., 2023). Blood biomarker discovery has been a topic of active research in AD during the last few years, and the following factors are related to AD pathology, the phosphorylated tau protein (p-tau), amyloid-ß (Aß42), and Aß42/Aß40, which have been in the limelight because these biomarkers are well related to the pathology of AD as seen in the CSF and imaging analysis. For instance, research has shown that increased plasma p-tau217 can differentiate patients suffering from Alzheimer’s from those diagnosed with other neurological disorders (Schneider, 2017; Delgado-Peraza et al., 2021).

#### 1.1.2 Prognostic capabilities

Apart from its diagnostic ability, BBBM takes a central stage in the prediction of outcomes. For example, high levels of p-tau217 have previously been reported to correlate with subsequent cognitive decline in patients with mild cognitive impairment (MCI), a disorder that is generally linked to AD. This predictive capability is central in helping identify people who are potentially at risk of getting dementia and early interventions that could act as the equalizers of the progress that the disease makes (Leuzy et al., 2022; Hampel et al., 2023). Moreover, the assessment of blood biomarkers can provide some added information about the disease-modulating effects of new drugs or lifestyle modifications during clinical trials. For example, observing the variations in biomarker values during therapy or disease development can be beneficial (Henriksen et al., 2014).

#### 1.1.3 Facilitating research and clinical trials

BBBM use in routine patient management can be enhanced by conducting clinical research through patient stratification. Therefore, it is easy for researchers to enrol people with pre-symptomatic AD or those with a considerable risk of developing the disorder to assess the effectiveness of interventions. This approach is especially related to the views of precision medicine when the treatment is chosen based on the biological characteristics of a patient (AlMansoori et al., 2024). Further, BBBM can be used as an outcome measure in clinical trials. This could result in improving the trial design and faster assessment of new therapies (Palmqvist et al., 2024).

### 1.2 Limitations and need for understanding biological determinants

A significant challenge is the variability of biomarker levels influenced by age, sex, genetics, comorbidities, and lifestyle factors. Age-related changes in plasma levels of Aβ and tau proteins can complicate direct assessment comparisons (Teunissen et al., 2022).

#### 1.2.1 Lack of specificity

Most of the BBBMs are not specific to AD high levels and may also be seen in other conditions like frontotemporal dementia or vascular dementia. Although DSM-IV is well-described in diagnosing dementia, but not specific in diagnosing AD and distinguishing it from other forms of dementia (Verheyen et al., 2021).

#### 1.2.2 Technical limitations

The existing conventional BBBM assays may not have the necessary accuracy or selectivity for clinical applications. There is a requirement for further improvement in the technologies that are directed towards better-advanced methods regarding the detection and reducing false negative/false positive ratio. The current advancements in methods of fixing higher sensitivity capable of measuring low concentrations of biomarkers are essential in increasing the accuracy of diagnostics (Hafkemeijer et al., 2016).

#### 1.2.3 Biological complexity

AD is complex, as the pathophysiology can be understood from the perspective of gene–environment interactions as well as a combination of genetic and lifestyle influences. Targeted analysis of biomarkers may fail to influence the development of certain diseases. Recognition of these determinants is highly relevant for better biomarker research and analysis (Rollo et al., 2016).

#### 1.2.4 Need for comprehensive understanding

To enhance the utility of BBBM in AD diagnosis and management, it is imperative to understand the biological determinants influencing these markers. Strong evidence exists suggesting that genetic makeup influences a person’s risk of getting AD; for example, individuals who carry the APOE ε4 allele have a higher risk of getting the disease. It might be valuable to look at the way genetic elements co-work with biomarker amounts to define the level of vulnerability. Epigenetic changes can alter genes without varying the sequences of the DNA. Scientific evidence also demonstrates that physico-chemical alterations play a major role in the development of AD from external influences like diet and stress, since the induction of epigenetic changes affects the genotype (Varesi et al., 2022). Perhaps comprehending these associations could expose fresh approaches to be used in intervention.

Inflammation has been involved with AD, and evaluating the relationship between inflammatory markers and neuroinflammation could yield important biomarker information. Lifestyle factors, including physical activity, diet, and smoking, can synergistically interact with biological factors associated with AD, contributing to the overall risk of developing the disease. For instance, how exercise, which decreases inflammation and increases cognition, alters biomarker levels would be useful for the possible prevention of frailty or AD in public health. Hence, BBBM for diagnosis, monitoring, and as well as treating AD has promising directions relative to both accuracy and efficiency. Nonetheless, several important limitations must be met when considering the biology of these markers. Subsequent studies should work towards the development of reference ranges of the various biomarkers while at the same time identifying genetic, epigenetic, inflammatory, and lifestyle determinants of the biomarkers. Thus, by incorporating such knowledge into clinical practice, we could improve our expertise in the early diagnosis of AD and design preventive and possibly curative approaches (Saha et al., 2017).

**Figure 1:**
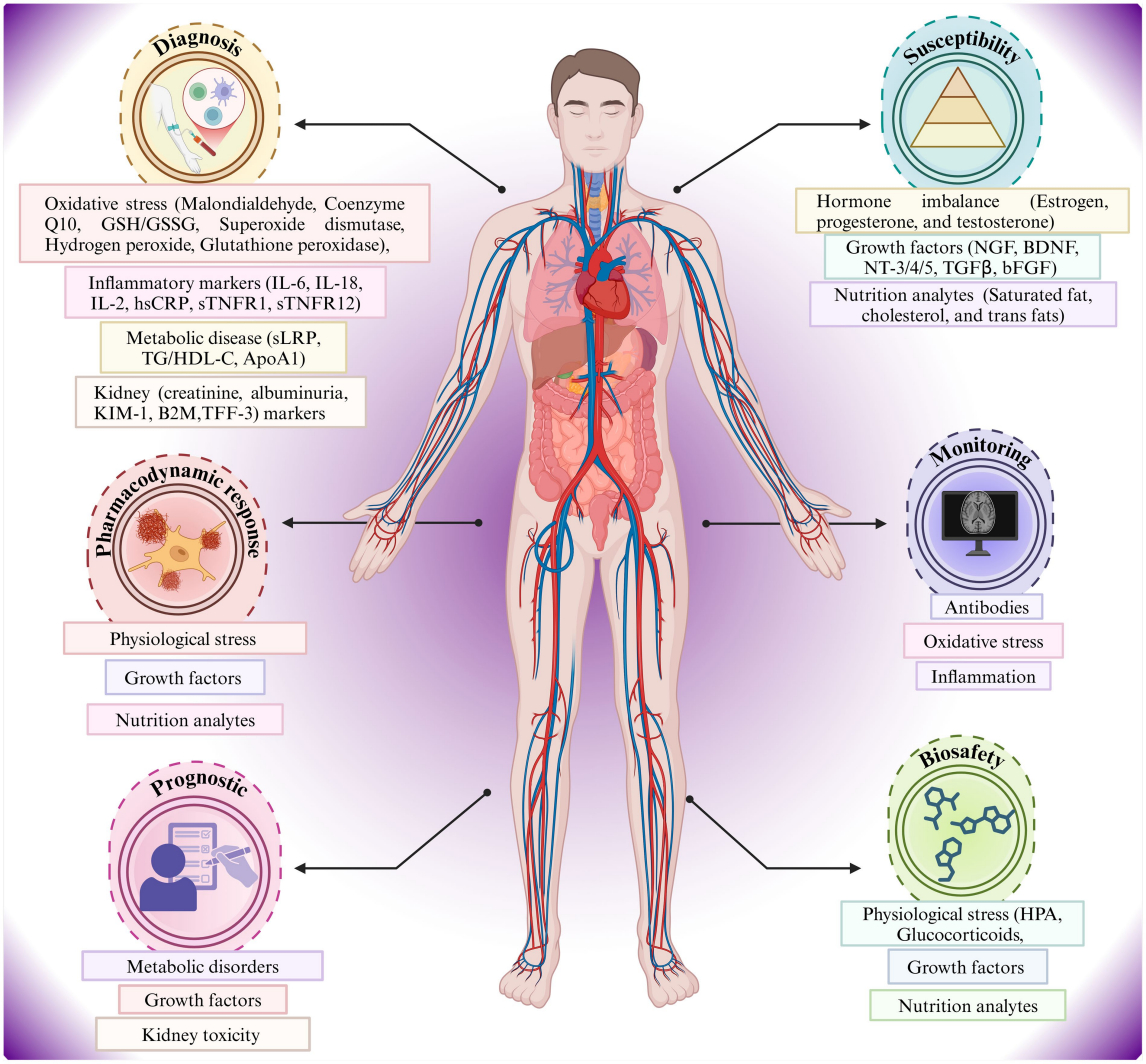
Importance of blood-based biomarkers (BBBM) in Alzheimer’s disease (AD).

Figure 1 portrays a relationship between the human body and the metabolic, inflammatory, oxidative stress, hormonal imbalance, and blood-based biomarker indicators of AD risk (Hampel et al., 2023). Diagnostic, susceptibility, monitoring, prognostic, and pharmacodynamic factors are shown in a systems-biology framework, indicating the effects of oxidative stress, metabolic disease, hormone imbalance, and nutritional analytes on the pathophysiology and individualized monitoring of AD (Teunissen et al., 2022). Key biomarkers include Aβ isoforms, phosphorylated tau, neurofilament light chain (NFL), and inflammatory proteins (Leuzy et al., 2022). Also, the figure highlights the multi-system and applicability of biomarker analysis in the management and risk stratification of AD.

### 1.3 Scope, novelty, and gaps addressed

While numerous prior reviews have separately examined the relevance of inflammatory processes, micronutrient status, or metabolic dysregulation in AD, these domains are rarely integrated to explain the variability observed in BBBM. The present review offers a new perspective by integrating cross-domain interactions – this review synthesizes the influence of nutrition, systemic inflammation, and metabolic health on each other and converges to alter biomarker expression, stability, and interpretability. Focusing on biomarker variability rather than absolute values - Unlike most prior literature, our emphasis is on biological and lifestyle determinants that shift biomarker levels within and between individuals, affecting diagnostic thresholds and longitudinal monitoring. The extent to which nutritional interventions modulate biomarkers independently of inflammation and metabolic state. Conflicting evidence on certain biomarker–risk factor associations, such as vitamin D status and cognitive decline. The lack of standardized reference ranges that adjust for physiological variability due to age, sex, APOE status, comorbidities, and lifestyle. Limited data on phenotypic differences in inflammatory and metabolic biomarker profiles between early-onset AD and late-onset AD. We outline an interdisciplinary model that links nutritional status, inflammatory load, and metabolic metrics with BBBM trends, incorporating multi-omics profiling and AI-driven analytics to improve predictive and diagnostic accuracy. Overall, this integration aims to support precision medicine, enabling biomarker interpretation to be tailored to the patient’s biological context. The novelty lies in combining diverse determinant domains into a single interpretative framework and mapping how their interplay influences biomarker trajectories across the AD continuum.

## 2 Materials and methods

### 2.1 Search strategy

This systematic literature review was conducted using a predefined search strategy to ensure a reproducible and transparent process. We searched multiple electronic databases, including PubMed, Embase, Medline, Google Scholar, Web of Science, Scopus, and Science Direct, for relevant articles published from January 2000 to the present. The search focused on identifying research publications, systematic reviews, and meta-analyses. We used a combination of keywords and Medical Subject Headings (MeSH) terms related to AD, BBBM, and the biological determinants influencing their levels. The search terms included “Alzheimer’s disease,” “AD,” “dementia,” “blood-based biomarkers,” “BBBMs,” “plasma biomarkers,” “serum biomarkers,” “Aβ,” “p-tau,” “NFL,” “nutrition,” “nutritional factors,” “vitamins,” “micronutrients,” “inflammation,” “inflammatory cytokines,” “metabolism,” “metabolic factors.” The overall collected data for this systematic review were processed in accordance with the PRISMA (Preferred Reporting Items for Systematic Reviews and Meta-Analyses) guidelines.

### 2.2 Study selection criteria

**Figure 2:**
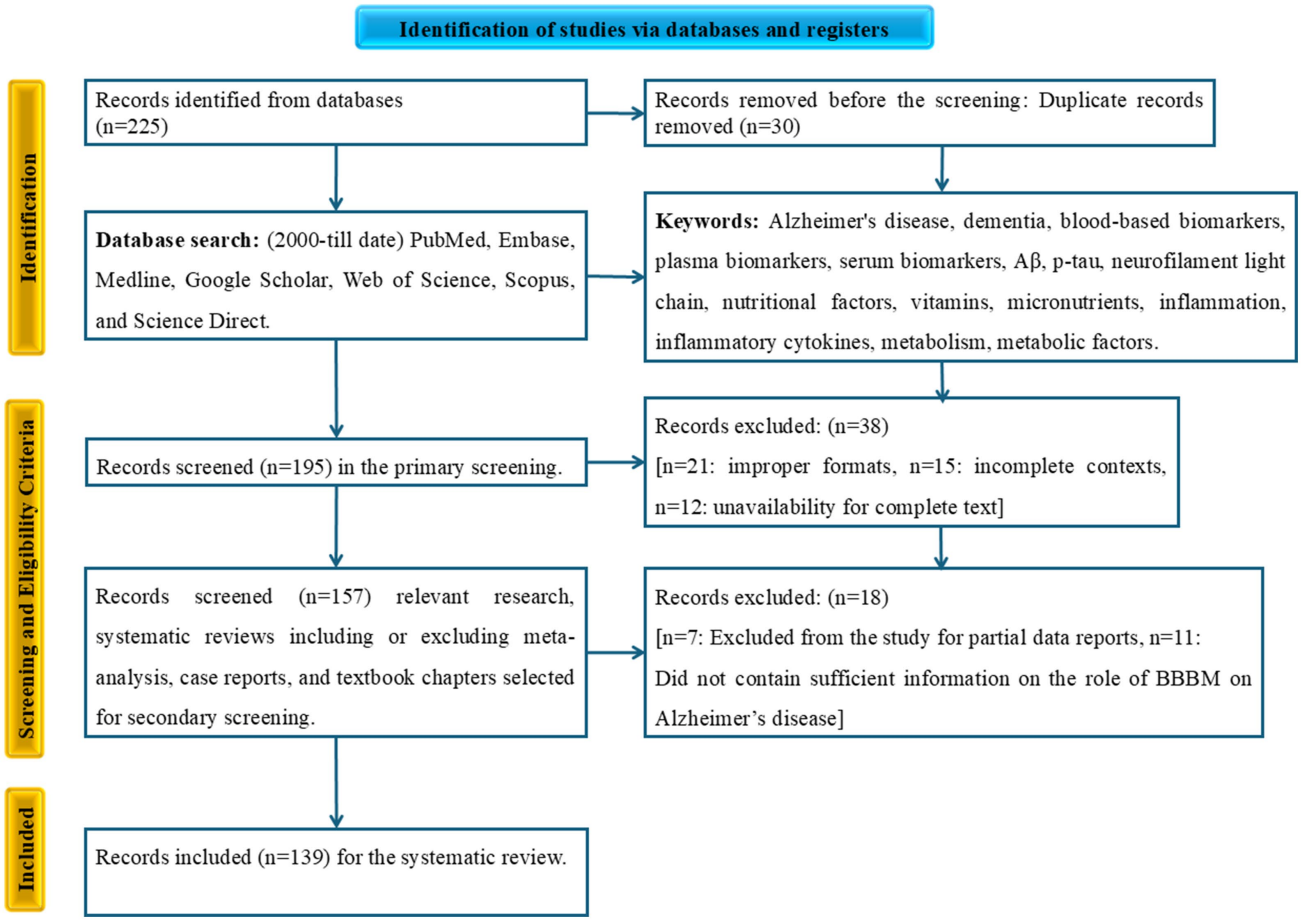
PRISMA flow diagram of study screening and selection.

All identified articles were evaluated based on a strict set of inclusion and exclusion criteria. We focused on studies that explored the relationship between blood-based biomarkers for AD and various nutritional, inflammatory, or metabolic factors in human subjects. An initial screening of records was conducted. After this, we identified and removed duplicate reports. Subsequently, a secondary screening was performed by assessing the titles and abstracts for relevance. Full-text articles were then retrieved and evaluated for eligibility. The final articles included in the review were those that met all inclusion criteria, such as being published in English, involving human subjects, and discussing the relationship between AD blood-based biomarkers and the specified biological determinants. Original research, systematic reviews, or meta-analyses. The exclusion criteria were animal studies or *in vitro* research, non-English articles, conference abstracts, editorials, or opinion pieces, and studies not focused on the specified biological determinants or blood-based biomarkers. The number of studies at each stage of this selection process will be detailed in the PRISMA flow diagram (Figure 2).

### 2.3 Data extraction

Data from the selected articles were systematically extracted and classified. Two independent reviewers extracted key information, including study design, population characteristics, the specific blood-based biomarker measured, and the nutritional, inflammatory, or metabolic factors investigated. Discrepancies were resolved by a supervisor. The extracted data were organized into categories to facilitate a comprehensive analysis. These categories included: articles describing the various blood-based biomarkers and their relevance to AD; articles focusing on specific biological determinants (e.g., nutrition, inflammation, metabolism) and their impact on these biomarkers; and articles describing the mechanistic link between the determinants and biomarker changes.

## 3 Results

### 3.1 Impact of nutritional factors on BBBM

The role of nutrition in determining and shaping health and disease concerning blood biochemistry is critically significant. These biomarkers can show the nutritional status of a human being, the metabolic activity occurring in the body, and the state of health of a person. This section shall investigate how nutrient factors affect BBBM (Pedlar et al., 2019).

### 3.2 Role of vitamins and micronutrients

Vitamins help subdue various activities in the body, which, in case of deficiency, result in various illnesses or diseases. An influence on BBBM seems to be evident since they alter the metabolic pathways, oxidative stress, and inflammation (Gariballa and Alessa, 2018).

#### 3.2.1 Vitamins

Vit. D is an essential nutrient for calcium metabolism and bone health, also supports the immune system, and helps regulate inflammation. Deficiency in Vit. D is closely linked with raised concentrations of certain inflammatory markers, including CRP and IL-6. Evidence from supplementation studies indicates that vitamin D, particularly in deficient populations, is associated with reductions in these inflammatory biomarkers, which may relate to a lowered risk of AD, though a direct preventive effect has not been consistently demonstrated (Picó et al., 2019). While several studies support a beneficial effect of vitamin D supplementation in modulating inflammatory markers relevant to AD risk, the evidence base is mixed. For instance, Martineau et al. (2017) found reduced risk of acute respiratory infections in vitamin D-deficient individuals, but large RCTs in generally healthy populations show no consistent cognitive or biomarker benefit (Martineau et al., 2017). The large-scale VITAL trial reported no significant reduction in major chronic disease endpoints, including cognitive decline, despite adequate dosing (Manson et al., 2019). A 2018 meta-analysis by Mazidi et al. concluded that vitamin D supplementation had no significant impact on CRP, IL-10, and TNF-α but significantly increased IL-6 levels in serum. The authors recommended larger randomized controlled trials (RCTs) with longer follow-up to clarify vitamin D’s effects on inflammation (Mazidi et al., 2018) A 2022 study by Krajewska et al. also showed vitamin D supplementation decreased CRP levels and influenced IL-10, though results vary by population and study design. They noted contradictory findings in the literature and highlighted the need for more targeted RCTs (Krajewska et al., 2022) Chandler et al. (2014) conducted a large, randomized placebo-controlled trial and reported no statistically significant changes in CRP, IL-6, IL-10, or sTNF-R2 with vitamin D supplementation in an African-American cohort, underscoring the complex relationship between vitamin D and inflammation (Chandler et al., 2014). Therefore, while mechanistic links to neuroinflammation exist, vitamin D’s role as a biomarker modifier should be interpreted cautiously. Vit. E helps to prevent oxidative stress and provides resistance to oxidation due to its antioxidant functions. Comparative analysis of different studies shows that the concentration of Vit. E in plasma is inversely related to malondialdehyde concentration as an indicator of oxidative stress. It also shows the necessity of Vit. E to safeguard the structural integrity of the cell and to help diminish inflammation (Capozzi and Bordoni, 2013). Bergin et al. (2021) conducted a systematic review and meta-analysis on Vitamin E supplementation’s effect on MDA, a biomarker of oxidative stress. They found that Vitamin E significantly reduced plasma MDA levels, supporting its antioxidant role, though there was considerable heterogeneity among studies, indicating the complexity of outcomes (Bergin et al., 2021) Wang et al. (2010) performed a double-blind trial with Vitamin E supplementation (100–300 IU/day) showing substantial reductions in oxidative stress markers including MDA by nearly 50% in plasma among metabolic syndrome patients, highlighting Vitamin E’s capacity to lower oxidative damage (Wang et al., 2010). Clinical trials on Vitamin E for AD prevention or slowing cognitive decline are mixed; some large trials found no significant benefit on cognition or AD progression, especially in early-stage patients, underscoring difficulties in translating antioxidant effects from experimental models to clinical success. This is an acknowledged challenge in interpreting antioxidant therapy outcomes. Farina et al. (2017), tested Vitamin E (2,000 IU/day) versus placebo in people with mild cognitive impairment (MCI) to see if it prevented progression to AD over 3 years in 516 participants. The study found no evidence that Vitamin E slowed progression or improved cognition, highlighting no significant benefit for MCI patients from Vitamin E supplementation (Farina et al., 2017). Dysken et al. (2014), A large trial in Veterans with mild to moderate AD showed that Vitamin E slowed functional decline by about 6 months compared to placebo, corresponding to a 20% slowing in disease progression per year. However, the effect on cognition specifically was not clearly significant, and Vitamin E outperformed memantine in this trial for functional outcomes (Dysken et al., 2014). Cochrane Review (2017) synthesized evidence from trials including one with 304 AD patients and one with 516 MCI patients. It concluded no clinically important cognitive benefit from Vitamin E in either group but did find moderate evidence that Vitamin E may slow functional decline in AD patients. No increased risk of serious adverse events or mortality with Vitamin E was found (Farina et al., 2017). Overall, Vitamin E lowers oxidative stress markers like MDA in plasma; evidence on its clinical efficacy for AD prevention remains inconclusive. The common vitamins that play an important role in homocysteine metabolism are vitamins B6, B12, and folic acid. Vitamin B12 plays a direct mechanistic role in one-carbon metabolism and myelin maintenance. Deficiency elevates plasma homocysteine, which induces oxidative stress, DNA damage, and activation of tau kinases (GSK3β), leading to increased phosphorylation of tau and higher circulating p-tau levels (Smith et al., 2018). *This cascade links an easily measurable nutritional biomarker to a core AD pathological marker. Also,* High homocysteine levels are associated with the presence of cardiovascular diseases as well as neurodegenerative disorders. Research revealed that a sufficient dosage of these vitamins may reduce homocysteine levels and, therefore, reduce the risk factors for associated diseases. Further, vit. B is an acknowledged participant in any synthesis of neurotransmitters, which are possibly involved in affectionate cognitive abilities (Khansari et al., 2009).

#### 3.2.2 Micronutrients

An efficient microelement encompassed by many enzymatic reactions and immune system functionality. The deficiency of zinc is associated with increased levels of oxidative stress and inflammation. The consumption of sufficient amounts of zinc has been reported to reduce the concentration of inflammation markers, including TNF-α and IL-6 (Xiao et al., 2024). In addition to its role in immunity, zinc appears to regulate T-cell function as a component of cellular immunity. Another micronutrient that can be considered very important is magnesium, since it also possesses anti-inflammatory properties. There’s evidence suggesting that increased magnesium consumption reduces hs-CRP and IL-6, which are both inflammatory markers. It was found that the deficiency of magnesium has been related to chronic diseases such as cardiovascular diseases and type 2 diabetes (T2DM; Au et al., 2015). Selenium was recognized for its antioxidant activity and for functioning in the form of thyroid hormones. Research has established that selenium intake results in a decrease in inflammation indices in patients with chronic diseases. Reduced selenium status has been related to increased levels of oxidant stress and inflammation (Greer, 2000).

### 3.3 Influence of macronutrients and dietary patterns

Specific macronutrients like carbohydrates, proteins, and fats are key dietary components that significantly influence blood-based biomarkers by affecting metabolism, inflammation, and overall health status.

#### 3.3.1 Carbohydrates

Refined carbohydrates are dietary sources with a high glycaemic index, like white bread and sugar-containing snacks, which have been associated with increased insulin resistance and higher levels of inflammation markers, including C-reactive protein (CRP). These foods cause a rapid rise in blood glucose levels and, thus, inflammation (Merino del Portillo et al., 2024). However, consumption of whole-grain products is known to be inversely related to inflammation, evident by low levels of inflammatory markers, because they are high in fibre and whole-grain phytonutrients. According to investigations, whole grains caused a decrease in levels of both IL-6 and TNF-α. The fibre in whole grains is also healthy for the gut since it increases the presence of healthy bacteria (Kusich, 2018).

### 3.4 Nutritional interventions and biomarker modulation

Nutritional changes, including dietary patterns or intake of nutrients, can potentially produce important changes in circulating markers of inflammation, oxidative stress, and overall health.

#### 3.4.1 Mediterranean diet

The Mediterranean diet emphasizes consuming whole foods such as fruits, vegetables, whole grains, legumes, nuts, and olive oil, along with moderate wine consumption, while limiting red meat and processed foods. According to scientific analysis, the Mediterranean diet is correlated to reduced inflammation. Oxidative stress is also brought down by the high antioxidant levels of fruits and vegetables in the diet. The research evidence indicates that there is enhanced cognitive performance among partakers of the Mediterranean diet than there is among partakers of the Western diet that is fraught with processed foods. This effect is because blood-based inflammatory biomarkers are becoming better (Bayer-Carter et al., 2011).

#### 3.4.2 Dietary approaches to stop hypertension (DASH) diet

The DASH diet focuses on reducing sodium intake while emphasizing fruits, vegetables, whole grains, lean proteins, and low-fat dairy. The DASH diet has been proven to reduce hypertension and also decrease other antigens like CRP. Unlike sodium, which is hypertensive, potassium is incorporated into the foods recommended by the diet (Filippou et al., 2020).

#### 3.4.3 Nutritional supplements

Nutritional supplements can also play a role in modulating biomarker levels. Omega-3 fatty acids have shown decreases in bid/current markers of inflammation, IL-6, and TNF-α in observational studies. Recent randomized controlled trial evidence shows that 12-month supplementation with combined omega-3 fatty acids significantly reduced plasma NFL levels - a blood marker of axonal injury - in individuals with mild cognitive impairment, suggesting a potential neuroprotective effect in early Alzheimer’s disease (Remoli et al., 2021). Especially significant in the potential carriers of chronic inflammation. Antioxidant vitamins like C and E lower certain measures of oxidant harm. However, research on their chronic illness-preventative effects remains inconclusive. More work is required to understand these molecules as modulators of biomarkers (Kalli, 2017).

**Figure 3:**
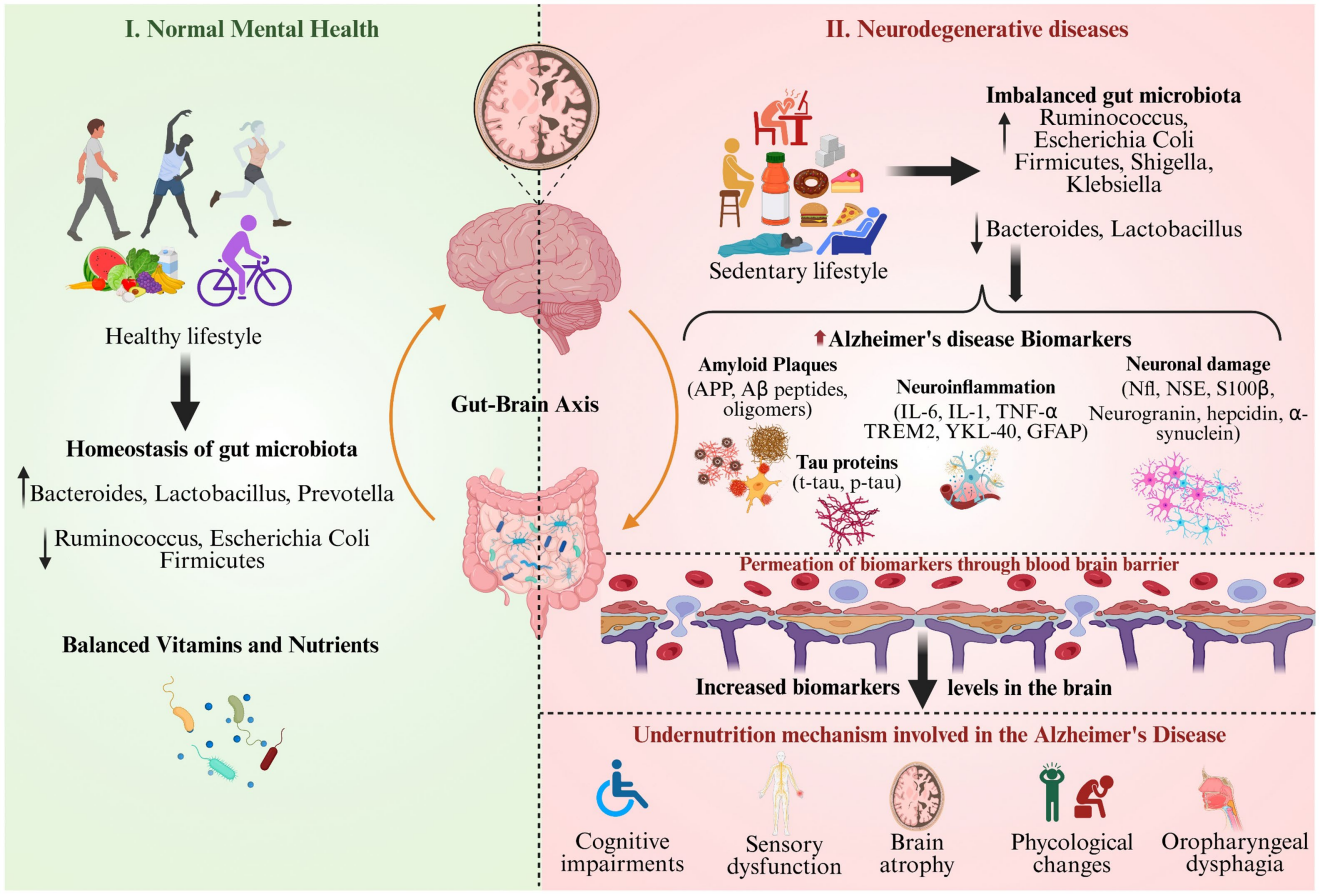
Nutritional factors responsible for biomarker modulation and Alzheimer’s disease progression.

Figure 3 compares healthy mental well-being, obtained through a balanced lifestyle and gut microbiota, with the development of neurodegenerative disease under an unhealthy and sedentary lifestyle (Inamdar et al., 2025a). On the left side, representing the normal metal health achieved with exercise and diet, which regulates the gut microbiome homeostasis, vitamin status, and brain integrity (Li et al., 2022). While on the right side depicts the effect of sedentary life and an unhealthy diet disturbs the gut microbiome levels, increases AD biomarkers and their permeability through the BBB, and impairs neuronal and cognitive function. The gut–brain axis, micronutrient status (e.g., vitamins D, E, and B12), and inflammatory modulation are highlighted as risk or protective factors for AD biomarker variability (Bayer-Carter et al., 2011). Also, the figure illustrates the gut-brain axis as a mediator, illustrating the impact of nutritional and microbial imbalance as risk factors for AD biomarker expression and undernutrition-induced cognitive and neurological impairment (Inamdar et al., 2025b).

## 4 Inflammation and its influence on biomarker levels

### 4.1 Role of systemic inflammatory mediators

#### 4.1.1 Cytokines as mediators

Subclinical inflammation usually takes place intracellularly, both in the cells of the tissues and the bloodstream all over the body. It can result from obesity, infections, and chronic diseases, among other things. It can occur either acutely or chronically, depending on its severity, and can be classified into different groups based on factors such as cytokines and chemokines, which represent a class of extracellular signalling molecules, are implicated in the regulation of brain function, being involved in neuroinflammation that is key to AD (Novoa et al., 2022; Wiatrak et al., 2023). Cytokine is mostly synthesized by activated microglial cells, and it is believed to play some role in the aggregation of Aβ plaque. IL-1β can also stimulate other inflammatory signals, thus prolonging inflammation. Even though IL-6 has both integrating and inhibitory consequences on inflammation, chronic spiked levels of IL-6 in AD are correlated with enhanced neuroinflammation and deteriorated cognition (Stewart and Beart, 2016).

#### 4.1.2 Chemokines and immune cell recruitment

Chemokines are special cytokines that inflame only the chemotaxis of nearby sensitive cells. In AD, chemokines orchestrate the immigration of immune cells to the affected parts of the brain. The effects of chemokine fractalkine (CX3CL1) include microglial activation, which has been established to be raised in both MCI and AD patients. Fractalkine is increased in AD and increases neuroinflammation at higher concentrations. CCL2 (MCP-1) attracts monocytes to the location of infection and inflammation. Previous studies have indicated that subjects with higher levels of CCL2 produce higher levels of microglial activation in AD (Stewart and Beart, 2016).

### 4.2 Chronic inflammation in AD pathophysiology

The current review asserts that AD is driven primarily by chronic inflammation. In the context of the inflammatory hypothesis, chronic inflammation is postulated to play an important role in mediating neuronal damage and compromised neurotransmission that results in cognitive decline.

#### 4.2.1 Microglial activation

Microglia, the critical cells belonging to the CNS tissue that defends the tissue in case of injury or certain illnesses. In AD, however, this activation becomes pathology and cannot be entirely controlled by the microglia themselves. The early activation of microglia might have a constructive function to aid in the degeneration of Aβ plaques. However, chronic activation results in phenotypic modification from the anti-inflammatory M2 to the pro-inflammatory (Heneka et al., 2015). M1 microglia secrete pro-inflammatory cytokines and reactive oxygen species (ROS) that can worsen neuronal damage while encouraging additional Aβ deposition. This transition is termed as an increased expression of other markers, including CD68 and CD11b (Long et al., 2022).

#### 4.2.2 Neurotoxic effects

Chronic neuroinflammation results in several neurotoxic effects, such as cytokine releases of pro-inflammatory cytokines resulting in synaptic breakdown and neuronal death (Singh et al., 2024). For example, IL-1β facilitates Aβ accumulation and at the same time activates the pathways leading to neuronal cell death. During chronic inflammation, the levels of ROS are higher, thus causing oxidative stress to the cell’s elements, including lipids, protein, and DNA This oxidative damage to the neurons is known to worsen the overall damage to the neurons.(Resende et al., 2007).

#### 4.2.3 Feedback loops

The relationship between Aβ accumulation and neuroinflammation creates a loop that perpetuates disease progression. The existence of Aβ plaques triggers the activation of microglial cells, which then secrete even more cytokines (Heneka et al., 2015). Cytokines further augment Aβ levels or lead to aggregation and thus give rise to inflammation as well as amyloid pathophysiology. Since neurons are also astoundingly sensitive to chronic inflammation, the latter unleashes containers packed with calls that recruit more immune cells to the site of the damage that leads to neuronal injury (Schindler et al., 2024).

### 4.3 Inflammatory biomarkers as co-factors in AD progression

Systemic inflammatory biomarkers have only recently been touted as markers of disease progression in AD. These biomarkers indicate active neuroinflammation and can potentially be used as targets for pharmacological treatment. Large-scale real-world validation shows that plasma p-tau217, when combined with other biomarkers including inflammatory measures, can identify Alzheimer’s pathology with over 90% diagnostic accuracy in both primary care and specialist settings, supporting its clinical utility beyond research environments (Palmqvist et al., 2024) There is emerging evidence of heterogeneity in inflammatory biomarker profiles across AD subtypes. For example, early-onset AD (EOAD) may present with lower peripheral CRP and IL-6 despite a high amyloid burden, whereas late-onset AD (LOAD) often shows elevated systemic inflammation (Leuzy et al., 2022). Similarly, individuals with amnestic MCI who progress to AD exhibit a different trajectory of plasma cytokines compared to those with non-amnestic MCI (Palmqvist et al., 2024) These subtype-specific patterns highlight the need to interpret inflammatory biomarkers within the clinical phenotype context.

#### 4.3.1 Identification of inflammatory biomarkers

Several inflammatory markers have been identified as potential biomarkers for AD. TREM2 receptor, known as the triggering receptor, expressed on myeloid cells 2, is present in microglial cells and has an active contribution in controlling inflammation. Higher levels of TREM2 mRNA have been linked to increased risk of AD. More recent evidence positing TREM2 variants associated with the risk of acquiring AD underlines its function in the pathophysiological process of the disease (Cai et al., 2022). Clustering affects the transport of lipids, and they also contain anti-inflammatory qualities. As seen in our results, the levels of clustering are higher in AD patients than in controls. It may be involved in the removal of Aβ aggregates from the brain (Ley, 2001).

#### 4.3.2 Chemokines as predictors

Chemokines such as CCL2 and fractalkine have shown promise as predictors for disease progression. High plasma concentrations of CCL2 are associated with the likelihood of dementia from MCI (Rojo et al., 2008). This implies that inflammatory patterns might affect the profile of cognitive degradation. Above all, higher fractalkine concentrations were linked with higher neuroinflammation in both MCI and AD patients; perhaps blood tests to determine fractalkine levels may offer information on the disease’s evolution (Zhao et al., 2020).

#### 4.3.3 Therapeutic implications

Understanding the role of inflammatory biomarkers opens avenues for therapeutic interventions. Anti-inflammatory strategies strive for the suppression of certain inflammatory processes to minimize the impact of neuroinflammation on the health of neurons. Similarly, blocking TNF-α or IL-1β action likely reduces the toxicity of Aβ without compromising the beneficial actions of these molecules (Leuzy et al., 2022). Table 2 provides a comprehensive overview of the major systemic and neuroinflammatory mechanisms implicated in AD, describing key inflammatory mediators - such as cytokines (IL-6, TNF-α), chemokines (CCL2/MCP-1, IL-8), and acute-phase proteins CRP- that drive pathological changes (Swardfager et al., 2010; Heneka et al., 2015). For each mechanism, the table specifies relevant biomarkers that are detectable in human blood or CSF, explains their clinical and experimental links to amyloid plaque formation, tau hyperphosphorylation, synaptic dysfunction, and neuronal loss, and outlines current or potential therapeutic interventions aimed at modulating these immune pathways (Leuzy et al., 2022). The evidence and implications summarized in the table are synthesized from leading primary research and meta-analyses to support clinicians and researchers in understanding how inflammation interacts with AD biomarker variability.

**Table 2:** Inflammation and its influence on biomarker levels leading to the pathophysiology of Alzheimer’s disease.

Inflammation	Description	Key inflammatory mediators	Impact on AD pathology	Therapeutic implications	Reference articles
Role of Systemic Inflammatory Mediators.	The cytokines in body fluids during infections or chronic diseases that come from systemic inflammatory responses can permeate the blood–brain barrier. This elicits neuroinflammation that may lead to neuronal degeneration and dementia in AD.	Cytokines are IL-6, TNF-α. Chemokines are MCP-1, IL-8. Acute-phase protein CRP.	Amyloid-beta deposition: higher deposition levels can be attributed to cytokine activity. Synaptic damage, there is evidence to show that cytokines worsen synaptic loss and, consequently, the functioning of the synaptic connection.	Anti-inflammatory treatments, including the drugs developed for the present invention, were designed to modulate the activity of IL-6 and TNF-α. Lifestyle modifications, such as changes in diet and exercise, were used as a disease-modifying approach to reduce systemic inflammation. Medications aimed at protecting the blood–brain barrier (BBB) were employed to prevent the diffusion of inflammatory mediators into the brain	Swardfager et al. (2010); Heneka et al. (2015)
Chronic Inflammation in AD Pathophysiology.	Chronic inflammation is characterized by constant stimulation of microglial cells that deploy neurotoxic mediators to the brain. However, this creates a toxic environment, leading to oxidative stress and neuronal degeneration.	Microglial activators such as IL-1β, IFN-γ. Oxidative stress markers like ROS and nitric oxide.	Microglial overactivation: When the microglial activation becomes chronic, the inflammation process is self-propelling. Neuronal death, long-term toxicity in the context of this project, is a continuous neuron loss.	Microglial inhibitors are substances that prevent chronic microglial activation (for example, minocycline) Some supplements, such as vitamin E, combat free radicals, which leads to antioxidant properties. Lifestyle interventions: Foods that contain antioxidants and fight inflammation-packed diets.	Holmes et al. (2009); Wyss-Coray and Rogers (2012)
Inflammatory Biomarkers as Co-Factors in AD Progression.	Blood, CSF, CRP, and IL6 are inflammatory markers associated with AD progression, correlated with brain volume loss and memory decrease. These biomarkers may also help tailor the patient to the respective therapy.	Biomarkers in blood/CSF, CRP, IL-1β, TNF-α Cellular markers in sTNFR (soluble TNF receptor), IL-6R (IL-6 receptor).	Cognitive decline is. Consequently, the faster the cognitive decline compared the higher the inflammatory biomarker levels. Brain atrophy includes biomarker levels reflecting a decrease in hippocampus and cortical volume.	These inflammatory biomarkers, including CRP, IL-6, and TNF-α, are associated with neuroinflammation in AD, which enhances neuronal loss and cognitive impairment. It may be therapeutic to target these markers in the hopes of diminishing inflammation and impeding the advancement of the disease.	Ritchie and Lovestone (2002)

## 5 Metabolic factors affecting biomarker variability

The interaction between the metabolism and the variability of biomarkers is a critical concept in the neurodegenerative processes of AD. The effects of glycaemic and lipid profiles, hormonal aspects, metabolism, and the role of metabolic syndrome in AD development are the three comprehensive analyses. Each section will provide a discussion of the biological reasons for biomarker variability.

### 5.1 Influence of blood glucose and lipid profiles

#### 5.1.1 Blood glucose variability and Alzheimer’s disease

Blood glucose levels are an important metabolic determinant of brain function. The elevated blood glucose level characteristic of T2DM increases the risk of dementia, including AD. Pulling on knowledge, it becomes possible to identify that T2DM patients have two times the risk of developing dementia compared with non-diabetics (Biessels and Whitmer, 2020). Some of the pathways through which this association occurs are insulin dysregulation and AGEs, which have neurotoxicity and encourage AD development due to increased amyloid aggregation and tau protein phosphorylation (Chun et al., 2022). Additionally, visit-to-visit variability was observed to have an association with dementia, based on previous research on blood glucose fluctuations. Analyzing data from a large sample containing over 32,000 patients across the country, the authors identified variations of the PV of metabolic parameters that predicted worse all-cause dementia and AD outcomes. This poses the possibility that the patterns of glycemia, the many rises and falls of glucose levels as much as the levels themselves, can cause cognitive decline (Ding et al., 2023). A recent large cohort study of >32,000 T2DM patients found that greater visit-to-visit variability in fasting glucose was independently associated with increased risk of dementia, including AD, over 8 years of follow-up. This supports the hypothesis that metabolic instability itself may contribute to biomarker fluctuation and brain pathology (Ding et al., 2023).

#### 5.1.2 Mechanisms linking blood glucose to neurodegeneration

The relationship between blood glucose levels and neurodegeneration can be explained through several mechanisms. Insulin resistance may also affect glucose transport into the brain’s cells and enhance a neuronal compromise in energy supply (Sharma et al., 2024). Such energy depletion can cause neuronal dysfunction and increasing the vulnerability of neurons to degenerative afflictions (de la Monte and Wands, 2008). High blood glucose concentration is known to upregulate oxidative stress by generating ROS. Oxidative stress is considered toxic to the neuronal cells and is pointed to for involvement in AD pathogenesis. Neuroinflammation is long-term high blood glucose can stimulate microglia—the brain’s immune cells - leading to neuroinflammation. They can also increase neuronal damage and feed into the inflammation that is the cause of cognitive decline (Brooks et al., 2005).

#### 5.1.3 Lipid profiles and neurodegeneration

Another important issue of research related to AD is lipid metabolism. Parent research using lipidomic has shown that people with AD are characterized by specific lipid patterns compared to the healthy population. Abnormalities in the levels of different lipid categories, including sphingomyelins, cholesterol esters, and phosphatidylcholines, were reported to be elevated in AD patients (Yoon et al., 2022). Lipidomic analysis in a 2022 observational study revealed that specific plasma sphingomyelins and phosphatidylcholines were significantly altered in AD patients compared to controls, correlating with CSF p-tau181 and Aβ42/40 ratio. These molecular lipid changes could partly account for variability in blood biomarkers driven by metabolic status (Yoon et al., 2022). These lipid changes may also indicate the pathobiological process associated with neuronal degeneration. Particular lipid species have been linked to genetic risk factors for AD. For instance, some specific risk polymorphisms, including the SNPs identified as intimately associated with AD risk, exhibit incomparably diverse effects on plasma lipid levels. This means that various genetic susceptibilities may interact with metabolic factors to affect AD progression (Sun et al., 2024). Abnormal lipid concentrations lowered by statins have been linked to augmented deposition of amyloid-beta in the brain. Cholesterol is important for synaptic transmission, but increased cholesterol levels alter lipid rafts, which are important for amyloid precursor protein (APP) processing (Kang et al., 2017). Omega-3 fatty acids have a direct effect on the brain; they are believed to suppress inflammation and oxidation within the brain. On the other hand, dietary SFA has been indicated to be related to a high risk of AD due to its inflammatory impact (Li et al., 2022).

#### 5.1.4 Implications for biomarker variability

Thus, patient characteristics that affect blood glucose and lipid variability may influence biomarkers used for diagnostic or prognostic purposes in AD. For instance, fluctuations in blood glucose levels can alter other variables, such as insulin-like growth factor-1 (IGF-1) or brain-derived neurotrophic factor (BDNF), which play roles in neuronal viability and synaptic remodelling. Changes in plasma lipid concentrations may impact molecular markers related to inflammation, C-reactive protein (CRP), or oxidative stress-malondialdehyde, which are elevated in patients with cognitive impairment dysfunction.

### 5.2 Hormonal regulation and metabolic health

#### 5.2.1 Insulin resistance and cognitive function

Insulin has conventional roles as a hormone primarily in regulating metabolism, but it is also crucial for the brain’s health. A particular type of insulin resistance involving the cell’s inability to respond to the hormone properly has been linked to cognitive impairment. In AD, insulin resistance fails to circulate within the brain and failure in of neuronal communication.

#### 5.2.2 Mechanisms linking insulin resistance to AD

Insulin signalling and amyloid-beta levels indicate that insulin may alter the status of this protein, which occupies a pivotal role in AD pathogenesis. Neuroinflammation is triggered through the release of some cytokines like interleukin 6 (IL-6) and tumour necrosis factor-alpha (TNF-alpha), which are products of inflammation and a key feature of insulin resistance, and they lead to cognitive impairment (Huang et al., 2020). Impaired synaptic plasticity is insulin signaling pathways that play an important role in the regulation of synaptic plasticity, which is needed for the learning and memory processes in organisms. Defects in this signalling pathway seemed to affect cognitive functioning(Hölscher, 2019).

#### 5.2.3 Thyroid hormones and brain health

Thyroid hormones are also important for metabolic regulation and intellect. The thyroid disorders have also been linked to the deterioration of cognitive function and dementia.

#### 5.2.4 Mechanisms linking thyroid hormones to cognitive function

Thyroid hormones T3 and T4 play a crucial role in brain development, with T3 deficiency impacting neurotransmitter metabolism, neurogenesis, and increasing oxidative stress, which is significant for the progression of Alzheimer’s disease. These hormones are particularly vital during the early stages of neural development, and a deficit can adversely affect cognitive abilities as reflected in IQ levels. Additionally, thyroid hormones regulate the synthesis of several neurotransmitters, notably serotonin, which is important for mood regulation, and dopamine, which is important for regulating motor control, motivation, and learning (Müller et al., 2022).

#### 5.2.5 Implications for biomarker variability

Hormonal dysregulation can significantly impact biomarker variability related to AD. Insulin sensitivity biomarkers lead to immobilization and changes in diet-induced insulin sensitivity, which may be reflected in fasting insulin or HOMA-IR indices, appreciated to be abnormal in AD candidates. Thyroid function biomarkers fluctuate in thyroid hormone levels and may affect risk factors associated with cognitive biomarkers such as BDNF or factors responsible for neuronal survival (Liao et al., 2021).

### 5.3 Metabolic syndrome (MET-S) and AD risk

#### 5.3.1 Defining MET-S

MET-S is a complex disorder that includes a group of related disorders such as abdominal obesity, hypertension, hyperglycaemia, and dyslipidaemia (Halagali et al., 2024). This syndrome is accompanied by raised rates of cardiovascular diseases and T2DM-both factors are considered to increase the chance of developing dementia (Frisardi et al., 2010).

#### 5.3.2 Components of MET-S

The components of MET-S include abdominal obesity, such as central obesity is a key risk factor for cognitive impairment according to metadata linking obesity to inflammation. Hypertension, while elevated blood pressure is associated with vascular dementia, is also a risk factor for AD generally. Many, and perhaps all, lipid profile dysregulations are directly linked to amyloid-beta disease progression (Paniagua, 2016).

#### 5.3.3 Mechanisms linking met-S to AD

Recent studies have established a significant association between MET-S components and the risk of developing AD. Inflammation: Low-grade chronic inflammation with obesity contributes to elevated cytokines, which are known to aggravate neurodegeneration. Insulin resistance-impaired glucose metabolism resulting from insulin resistance, typical of metabolic syndrome, is believed to cause neurodegeneration directly (Inamdar et al., 2025b). Vascular dysfunction and metabolic syndrome are associated with various vascular diseases that might affect cerebral blood flow and cause ischemic injury, the major precondition for the formation of cognitive dysfunction (Frisardi et al., 2010).

**Figure 4:**
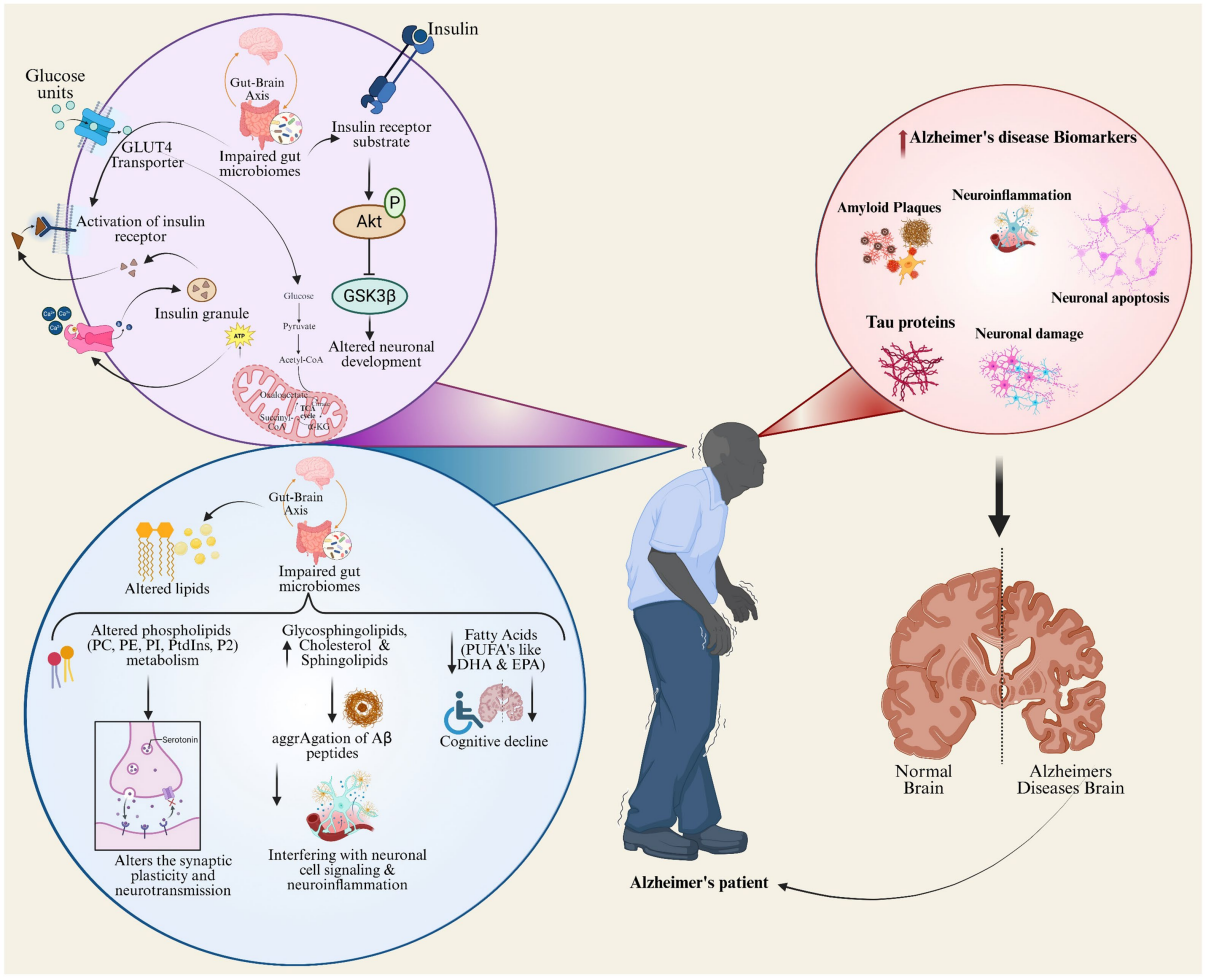
Metabolic syndrome and confounding factors responsible for Alzheimer’s disease progress.

Figure 4 depicts the lipid metabolism and insulin signaling pathways integration as a major underlying cause of AD pathophysiology. This pictorial representation of glucose metabolism connects to the lipid alterations, gut-brain axis impairments, and hormonal signaling dysregulation leading to neuronal dysfunction and cognitive decline by interacting with inflammatory processes to accelerate amyloid plaque formation, tau aggregation, and neuronal loss (Frisardi et al., 2010). Depicts key AD biomarkers within the context of metabolic dysregulation (Li et al., 2022). This schematic depicts the impairment in insulin signaling and gut microbiome disruption impair neuronal development; disturbed lipid profiles and inflammation worsen amyloid plaque formation, tau aggregation, and neurodegeneration. The central illustration with an Alzheimer’s patient establishes a link to the brain changes and systemic processes and presence of key AD biomarkers like amyloid plaques, tau proteins, and neuroinflammation, thus showcasing succinctly the multifactorial biological contributors to AD pathology.

## 6 Mechanistic pathways between nutrition, inflammation, and metabolism in ad biomarker expression

### 6.1 Interconnected pathways and clinical implications

Nutrition and inflammation, as well as their connection to metabolism, are rather complex and play a crucial role in AD. Overall, these factors integrate through several biological mechanisms that affect the levels of biomarkers relevant to AD. Deficiencies in key micronutrients such as vitamin B12, vitamin D, vitamin E, and folate disrupt central metabolic cycles, particularly one-carbon metabolism-thereby increasing homocysteine levels, promoting oxidative stress, and facilitating aberrant tau phosphorylation and Aβ dysregulation. Elevated homocysteine, resulting from impaired methylation cycles, is a recognized risk factor for tauopathy and shifts in plasma Aβ42/40 ratios. Additionally, insufficient antioxidant vitamins further exacerbate oxidative stress, destabilizing neuronal health (Xu Lou et al., 2023). Chronic inflammation, marked by elevated circulating cytokines like IL-6 and TNF-α, provokes microglial activation and neuroinflammation. These processes increase neuronal injury and are reflected by heightened blood levels of biomarkers such as NFL chain, p-tau, and Aβ. Notably, systemic cytokine signaling affects blood–brain barrier permeability, amplifying neural insult and peripheral biomarker release (Park et al., 2025). Metabolic syndrome-characterized by central insulin resistance, disrupted lipid metabolism, and accumulation of advanced glycation end-products (AGEs)-impairs neuronal glucose homeostasis, enhances oxidative injury, and accelerates amyloidogenic and tauopathic processes. Altered lipid profiles and hyperglycemia further contribute to vascular dysfunction and neurodegeneration, modulating levels of AD biomarkers in circulation (Więckowska-Gacek et al., 2021). For insight, the tryptophan-kynurenine pathway, dysregulation increases neurotoxic metabolites (quinolinic acid) and links peripheral inflammation with AD biomarker dynamics (Liang et al., 2022). Disrupted tyrosine pathway & oxidative Stress, leading to altered tyrosine metabolism, is a nodal point connecting peripheral metabolic disturbances, enhanced oxidative injury, and altered plasma biomarkers. Mitochondrial dysfunction, due to both nutrient deficits and insulin resistance, converges on mitochondrial pathways, diminishing cellular bioenergetics and affecting biomarker profiles, and leading to the progression of AD.

### 6.2 Emerging blood-based biomarkers for early and specific detection of Alzheimer’s disease

Recent studies in 2023–2024 have demonstrated the clinical feasibility and robust diagnostic performance of plasma p-tau assays, particularly p-tau217 and p-tau231. Ashton et al. evaluated a novel commercial plasma p-tau217 S-PLEX assay with excellent technical performance, achieving an area under the curve (AUC) of 0.98, distinguishing AD patients from controls, outperforming p-tau181, and aligning well with CSF and PET biomarkers (Kivisäkk et al., 2024). Another large cohort study validated the utility of plasma p-tau217 as a screening tool with the potential to reduce confirmatory testing by approximately 80% (Ashton et al., 2024). Head-to-head comparisons of multiple plasma p-tau assays confirmed the superiority of p-tau217 for detecting abnormal amyloid status and predicting progression, supporting its clinical adoption (Janelidze et al., 2023). Alongside assay advancements, digital cognitive phenotyping tools are evolving and show promise for integrating objective cognitive metrics with biomarker data to improve early detection and monitoring. Concurrently, global harmonization efforts by international consortia are advancing standardized biomarker protocols and reference materials, essential for consistent clinical application across populations and platforms.

Beyond the well-established biomarkers Aβ, p-tau, and NFL, recent research has illuminated a range of emerging candidates that hold promise for earlier detection and greater disease specificity. Exosomal microRNAs (miRNAs), which are circulating exosomes carrying brain-derived miRNAs, have emerged as minimally invasive indicators reflecting neuronal health and pathophysiological processes in AD. Specific miRNA signatures linked to synaptic function and neuroinflammation have been proposed as early predictive biomarkers, potentially preceding detectable changes in classical markers (Alhenaky et al., 2024). Advances in high-throughput plasma proteomic technologies have identified novel protein candidates in plasma associated with synaptic integrity, neuroimmune signalling, and neurodegeneration. For example, recent studies report the ratio of synaptic proteins YWHAG and NPTX2 in CSF and plasma as a strong indicator of cognitive resilience and disease progression risk, independent of classical amyloid and tau pathology (Jiang et al., 2022). Comprehensive metabolomic profiling has revealed disturbances in pathways such as lipid metabolism, amino acid turnover, and energy metabolism, which correlate with AD stages and cognitive decline. These metabolic fingerprints in blood can complement traditional biomarkers to better capture disease heterogeneity and progression (Yu et al., 2023). Integration of these emerging biomarkers with established panels and multi-omics approaches offers a promising future direction toward more sensitive, specific, and earlier diagnosis of AD, as well as personalized therapeutic monitoring.

#### 6.2.1 Nutrition and its role in inflammation

Inflammation is a natural phenomenon regulated by nutrition in the human body. Diets such as antioxidants, omega fatty acids, and polyphenol diets have been associated with successful moderation of inflammation and oxidative stress, which defines the major indicators of AD pathology (Li et al., 2022). On the other hand, there are increased saturated fats and sugars that are known to perpetuate the inflammation processes. Mediterranean diet studies have confirmed that traditions of healthy diets, which include food of the Mediterranean style with protected fruits, vegetables, whole grains, fish, and fluid fats like olive oil, diminished the risk of impaired impairment and inflammatory marker levels in a civic population (Scarmeas et al., 2006). This dietary pattern may improve neuroprotective factors by lowering these indices of inflammation, such as IL-6 and TNF-α. The Western diet is, on the other hand, a diet that is characteristic of Western countries, associated with a high intake of processed foods and refined sugars, and has been associated with increased systemic inflammation and amyloid-beta levels, which are essential to the AD disease process. The statistics have shown that high-GI foods can increase blood sugar levels and activate insulin resistance and neuroinflammation.

While numerous nutritional and lifestyle interventions are proposed as modulators of AD biomarker expression, current evidence varies in strength and magnitude. Meta-analyses indicate a small but significant beneficial effect of dietary patterns, such as Mediterranean and ketogenic diets, and specific nutrient supplementation (omega-3 fatty acids, vitamins D and B12) on cognitive outcomes and AD-related biomarkers, including Aβ and p-tau proteins (Xu Lou et al., 2023). For insight, a systematic review and meta-analysis reported a modest effect size (β = 0.11) for diet adherence reducing AD biomarker burden (Josephs et al., 2019). Observational studies report associations between higher intake of nutrients such as vitamins B12, D, and omega-3 polyunsaturated fatty acids with lower cerebral amyloid burden measured by PET imaging (Zhao et al., 2025). However, many studies remain limited by small sample sizes, heterogeneous study designs, and observational or cross-sectional nature, highlighting the need for more longitudinal randomized controlled trials to conclusively establish causal effects. Furthermore, the clinical utility of these interventions as reflected by biomarker modulation has not been fully validated, warranting cautious interpretation and explicit acknowledgment of these limitations. Altogether, while nutritional and lifestyle modifications show promise as adjunctive strategies to modulate AD pathophysiology, current evidence should be interpreted conservatively, emphasizing ongoing research needs to quantify effect sizes and validate biomarker changes as clinically meaningful.

#### 6.2.2 Metabolism and inflammation

The metabolism process is interwoven with known processes for inflammation. Defective insulin signalling, central to metabolic syndrome, has been linked to increased production of inflammatory cytokines, which are toxic to neurons. Insulin resistance is the preloading of the isolated rat kidneys with captopril reduces blood pressure and inhibits the synthesis of angiotensin I by 80% and angiotensin II by 60%. Raised insulin concentration can also induce neuroinflammation by stimulating microglial cells—the primary immune cells in the brain. Adipose tissue inflammation is Central obesity also begets a chronic low-grade inflammatory state by the release of inflammatory adipokines like leptin and resistin. It is worth stressing that this inflammatory state may negatively affect neuronal communication and stimulate amyloid-beta deposition (Hölscher, 2019).

#### 6.2.3 Biomarkers of inflammation in AD

Several biomarkers reflect the inflammatory state in individuals with AD. High levels of CRP have been directly linked to accelerated cognitive decline and are therefore used as an inflammation biomarker (Koyama et al., 2013). Interleukin-6 (IL-6) is a pro-inflammatory cytokine that has been linked with neurodegeneration. There have been suggestions that elevated levels of serum IL-6 are related to higher levels of amyloid in the brain (Swardfager et al., 2010).

### 6.3 Integrative approaches to biomarker analysis

Inclusive biomarker analytic strategies include the use of genomic, proteomic, metabolomic, and clinical data sets to achieve a detailed picture of disease mechanisms. It makes this methodology especially useful in multifaceted conditions like AD.

#### 6.3.1 Multi-omics integration

The integration of multi-omics data allows researchers to capture the complexity of biological systems involved in AD. Recent data from genome-wide scans have revealed many SNP markers that are linked to the risk of AD. For example, recent genetic variants close to the APOE gene are already known to impact the hazard of developing late-onset AD (Hollingworth et al., 2011). Imaging of proteomics data can be used to detect various proteins in the disease process of AD pathology. For instance, amyloid precursor protein processing has been associated with lipid metabolism because of genetic changes. Metabolomics thus helps reveal those metabolic dysfunctions that are implicated in AD. Research works suggest paramount metabolite changes in individuals experiencing some form of cognitive loss, and some of the affected metabolites concern energy metabolism (Lauer et al., 2021).

#### 6.3.2 Systems biology approaches

Systems biology approaches utilize computational models to analyze complex interactions between biological components. Crossing paths of omics data allows for building interaction networks to detect such nodes to regulate the disease that may have the potential for successful targeting in AD (Zhang et al., 2020). Machine learning algorithms can be utilized in identifying biomarker signatures since these techniques can discern patterns within big data that might not be easily recognizable by statistical methods alone, and are possible to use by amassing data of various types for constructing prognostic models on the development of diseases (Huang et al., 2023). Integrative systems biology and multi-omics approaches offer dynamic, holistic mapping of how nutritional, metabolic, and inflammatory signals intersect to shape AD pathogenesis. Omics-based network biology enables delineation of direct, indirect, and feedback relationships among these multifactorial determinants and BBBM (Leventhal et al., 2025) Recent systems biology studies have produced network diagrams illustrating how nutritional factors regulate metabolic fluxes, immune cell activation, and ultimately, the release and modification of AD biomarkers (González-Domínguez et al., 2021). These models allow for testable causal inference, simulating the effects of dietary interventions, anti-inflammatory agents, or micronutrient supplementation on biomarker profiles and clinical outcomes (Castrillo et al., 2018). Incorporating these frameworks advances the field from correlation toward causality by mechanistically modeling the impact of perturbations and integrating findings across genomic, proteomic, and metabolomic layers.

#### 6.3.3 Integration of multi-omics and AI-based analytical strategies in Alzheimer’s disease research

Integrative biomarker analysis has significant clinical implications for AD. In early diagnosis, clinicians may be able to diagnose AD at a much earlier stage if they determine specific biomarkers that can reflect inflammation and metabolic dysregulation. Personalized treatment strategies, modifications in biomarkers because of genetic or lifestyle differences, explain that a few individuals may need anti-inflammatory intervention or optimization of their metabolic profile (Melzer et al., 2020). Recent advances in integrating multi-omics datasets with artificial intelligence (AI) and machine learning (ML) approaches have begun to substantially enhance our understanding of AD pathophysiology, biomarker discovery, and patient stratification. For instance, frameworks such as PRISM-ML integrate transcriptomic and genomic data from large multi-region post-mortem brain cohorts to identify tissue-specific molecular signatures, employing interpretable models such as Random Forests with SHapley Additive exPlanations (SHAP) to reveal hub genes and biological pathways with therapeutic potential (Cardillo et al., 2025). Multi-modal fusion models that combine proteomic, metabolomic, neuroimaging, and cognitive data have achieved over 90% diagnostic accuracy for differentiating AD from related dementias, using algorithms such as CatBoost with optimized hyperparameters and decision-level fusion strategies (Hassan et al., 2025). Moreover, graph neural networks (GNNs) that embed biological network priors have improved both the predictive power and interpretability of multi-omics classifiers, enabling causal inference and drug repurposing pipelines (Tripathy et al., 2025). Despite this progress, challenges remain-including batch effects, cohort heterogeneity, and limited prospective validation-which currently constrain clinical translation. Addressing these limitations through standardized pipelines, cross-cohort validation, and integration of emerging biomarkers will be critical to realizing the full potential of AI-driven multi-omics for precision medicine in AD.

### 6.4 Cross-talk between nutrition, inflammation, and metabolism: implications for biomarker variability

The biological determinants discussed in this review do not act in isolation; instead, they form interconnected pathways that jointly influence BBBM levels in AD. Dietary patterns directly affect metabolic and inflammatory status - for example, excess intake of saturated fats and refined sugars promotes obesity, insulin resistance, and dyslipidaemia, which in turn amplify pro-inflammatory cytokines such as IL-6 and TNF-α. This low-grade systemic inflammation can accelerate amyloid aggregation, tau phosphorylation, and subsequent neuronal damage, thereby shifting levels of Aβ42/40, p-tau, and NFL chain in circulation (Frisardi et al., 2010).

Conversely, anti-inflammatory dietary patterns - such as the Mediterranean or DASH diet - may improve lipid and glucose profiles, reduce oxidative stress, and suppress pro-inflammatory mediators, supporting biomarker stability over time. Nutrient deficiencies (e.g., vitamin B_12_, D, E) can interact with metabolic disorders by exacerbating homocysteine accumulation, oxidative injury, and microglial activation, further destabilising biomarker readouts (Li et al., 2022).

In metabolic syndrome, the convergence of hyperglycaemia, insulin resistance, hypertension, and dyslipidaemia creates a milieu in which inflammatory and metabolic pathways perpetuate each other. This “vicious cycle” can cause dynamic biomarker fluctuations unrelated to short-term disease progression, complicating longitudinal interpretation. Breaking these cycles through integrative interventions and combining dietary optimization, metabolic control, and inflammation management may reduce biomarker levels and improve their diagnostic and prognostic value.

Overall, understanding these cross-domain interactions is essential for precision biomarker interpretation. Future studies should focus on modelling these interactions using multi-omics data and machine learning, enabling personalised biomarker thresholds that consider the patient’s nutritional, inflammatory, and metabolic context (Hampel et al., 2023).

### 6.5 Consideration of confounding factors affecting blood-based biomarkers

The translational importance of blood-based biomarkers in Alzheimer’s disease diagnosis and prognosis, it is essential to account for confounding factors such as age, sex, ethnicity, comorbidities, and medication use, as these variably influence biomarker levels and the accuracy of diagnostic thresholds (Inamdar et al., 2025d). Large cohort studies (BioFINDER) have identified variables like creatinine and body mass index (BMI) as significant modulators of plasma NFL, glial fibrillary acidic protein (GFAP), and p-tau, although these analyses show only modest impact on diagnostic performance (Pichet Binette et al., 2023). Age and sex are consistently adjusted due to well-characterized influences on biomarker variance (Grande et al., 2025). Ethnic diversity and comorbidities, particularly renal function and systemic inflammatory states, further modulate biomarker concentrations and require consideration in diagnostic algorithms to ensure broad applicability (Kurz et al., 2025). A thorough understanding of these factors is crucial for interpreting biomarkers results in clinical practice and for developing robust, context-sensitive diagnostic thresholds.

## 7 Implications for personalized medicine (PM) in AD

PM concept applied to AD has great potential for enhancing the diagnosis and treatment, as well as patients’ outcomes. This approach focuses on the customization of the healthcare process as well as biomarker analysis and diagnostics. This document will delve into two key areas, such as adapting biomarker meaning to individual behavioral patterns and the suggestions for precision early recognition and tracking.

### 7.1 Tailoring biomarker interpretation to individual profiles

#### 7.1.1 Understanding biomarkers in AD

Biomarkers can be defined as referring to biological markers that help in giving out important information about the disease status. In the context of AD, several types of biomarkers have been identified, including Genetic Biomarkers. Other factors that are linked to the disease include the APOE ε4 allele, which increases the likelihood of having AD severalfold. Knowledge about a patient’s genetic constitution may be useful in evaluating his/her risk and the preventive measures to be taken (Forloni, 2020). Neuroimaging biomarkers are molecular imaging methods, such as PET scans, that provide a possibility of visualizing amyloid plaques and tau tangles, which characterize AD. Such imaging biomarkers can thus describe the disease’s advance even at stages when patients show no signs of it at all (Ahmed et al., 2023). Cerebrospinal Fluid (CSF) Biomarkers are commercially available assays that can be performed on CSF, consisting of amyloid-beta protein and total phosphorylated tau measure neurodegeneration. These biomarkers are vital in distinguishing between AD and other dementias (Gauthier et al., 2018).

#### 7.1.2 Personalized interpretation of biomarkers

The interpretation of these biomarkers must be individualized, taking into account various factors such as age and cognitive baseline, suggesting that amyloid plaques could be unrelated to memory and other cognitive functions for older people. Hence, knowing the specific mental capability of a certain person goes a long way in evaluation (Ganesh et al., 2023). Comorbid Conditions with other associated diseases are different in their biomarkers, which call for a differential approach to diagnosis and management (Hangel et al., 2024). Clinical presentation variability in these AD may present heterogeneously; therefore, biomarker-enforced analysis should consider pathological presentations that do not fit the currently used clinical classification (Kim et al., 2020).

#### 7.1.3 Innovative approaches to biomarker analysis

Recent advancements have led to innovative methods for analyzing biomarkers that enhance PM approaches like Multiplexed sensing technologies. The latest techniques make it possible to explore several AD biomarkers at the same time to assess the overall state of a patient (Inamdar et al., 2025c). For example, a recent study showed that a sensor array based on a carbon nanotube was capable of identifying critical AD biomarkers with considerable sensitivity and specificity, enabling the separation of AD patients from healthy individuals (Craig-Schapiro et al., 2011; Assfaw et al., 2024). BBBM leads to a high-risk, low-invasive blood marker test that has been created at the University of Pittsburgh that shows over 100 biomarkers linked with AD. This test could dramatically alter the clinical approach to risk assessment before the manifestation of cognitive first symptoms (Forloni, 2020; Keshavan et al., 2021). In a pragmatic screening of a general older adult cohort (n ≈ 500), plasma p-tau181 and Aβ42/40 ratio accurately identified individuals with abnormal amyloid-PET scans (Keshavan et al., 2021). This direct population-based evidence strengthens the case for implementing validated blood biomarker protocols in real-world risk assessment.

### 7.2 Opportunities for precision diagnostics and monitoring

Currently, the picture of Alzheimer’s diagnostics is far from being constant—further progress in technology and our knowledge regarding the pathophysiology of Alzβ-pathology in patients disease has been observed lately (Gauthier et al., 2018). Precision diagnostics is a concept that will enable doctors to diagnose diseases accurately and address the treatment regimen according to the patient’s characteristics (Guest et al., 2020).

#### 7.2.1 Advanced diagnostic techniques

A few diagnostic techniques have been developed, including genomics profiling is a current next-generation sequencing technology that helps to achieve TWGS of the complex genetic variation implicated in AD pathogenesis (Pauwels and Boer, 2024). This information can be useful to develop individual treatment approaches based on patients’ genetic characteristics. Biomarker estimation allows for greater accuracy; instead of using a single marker, various range of biomarkers can be utilized in clinical practice (Behl et al., 2022). Thus, the novel multi-biomarker strategies can offer better discrimination between AD and other dementias as well as offer prognostic data on the disease (Khoury et al., 2017). A mathematical model is the AD Biomarker Cascade (ADBC), constructed to predict the disease progression of actual patients using biomarker data. Due to this, they espouse treatments that accord with an individual’s disease progression model (Assfaw et al., 2024).

**Figure 5:**
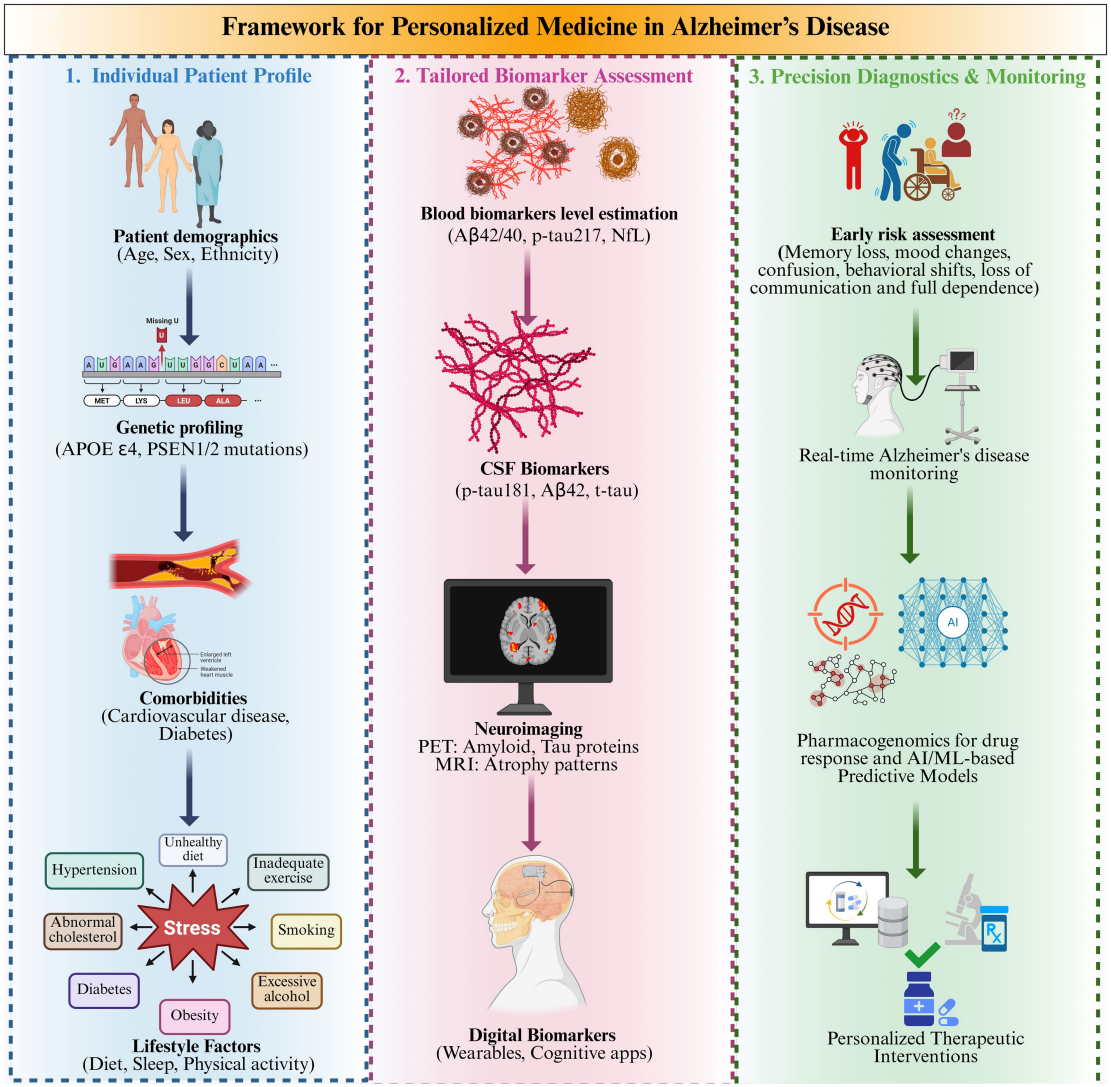
Framework for personalized medicine via monitoring levels of blood-based biomarker levels in Alzheimer’s disease.

Figure 5 is sectioned into a three-part framework for precision medicine in AD. Initiating with inductive profiling of individual patients, including their demographics, genetics, comorbidities, and lifestyle factors, continuing to develop customized estimation of biomarker levels via blood, CSF, neuroimaging, and digital markers, and finally finishing with advanced diagnostic and monitoring approaches (Hampel et al., 2023). The integration of AI/ML, pharmacogenomics, and digital health tools for risk assessment and therapeutic interventions is illustrated in the rightmost panel (Assfaw et al., 2024). Combining comprehensive insights into biomarker profiling could provide individualized disease tracking and customized treatment of AD.

#### 7.2.2 Monitoring disease progression

Precision medicine also encompasses monitoring disease progression through various innovative strategies, such as regular biomarker assessment can be measured repeatedly over time using blood tests or CSF analysis to determine change over time and the potential benefit of treatment or disease progression. Digital health technologies like smart gadgets and mobile applications measure cognitive functioning and daily activities, and the results are used to modify treatments accordingly.(Ahmed et al., 2023; Juganavar et al., 2023). This seems to be done through longitudinal designs, where the same patient sample is followed for a long interval. Often, the patients agree to undergo these longer investigations. In this way, the researchers can understand how biomarkers, proteins, or chemicals that indicate disease in the body are altered and can then correlate these changes with the well-being of patients. This enables researchers to determine the different connections of these biomarkers to the disease’s progression. This information is important for enhancing the accuracy of the algorithm used for treatment plans (Rosas et al., 2020; Poulet and Durrleman, 2023).

#### 7.2.3 Challenges in implementation

Despite the potential benefits, several challenges must be addressed when implementing PM in Alzheimer’s care ethical considerations regarding the action with genetic information are ambiguous about privacy and discrimination due to the genetic predisposition of the person (El-Sappagh et al., 2021; Assfaw et al., 2024). Access to technology is inequalities in the enrolment of patients into national diagnostics coordinate frameworks that hinder the practice of precision medicine across populations (Khoury et al., 2017; Keshavan et al., 2021). Integration into clinical practice has to train healthcare professionals on integrated functioning and also establish protocols for biomarker assessment (Pascoal et al., 2024).

## 8 Clinical trials

### 8.1 The role of biomarkers in clinical trials

Biomarkers are biological signs that give specific information on physiological and pathological conditions in response to treatment. In the context of AD, biomarkers are essential for several reasons, such as accurate clinical diagnosis of AD, as a lack of well-developed clinical assessments can miss the mark by 10 to 15 percent. Biomarkers enhance the diagnostic resolution by presenting ailment indicators, for example, amyloid plaques and tau tangles, which characterize AD (Owen et al., 2023). Patient selection is successfully incorporated and engenders the ability to enroll more specific subjects in clinical trials concerning biomarkers. In this way, the researchers can limit study subjects to only those with the relevant pathologies that make up AD, thereby increasing the chances of detecting treatment outcomes (Hansson et al., 2023). Monitoring treatment effects, such as biomarkers, facilitates the evaluation of pharmacodynamic treatment outcomes compared to simple neuropsychological tests. For example, increases or decreases in phosphorylated tau (p-tau) or NFL chain in blood tests can show how effective a therapy is at a biomolecular level (Henriksen et al., 2014). Supporting regulatory approval gives the latest clearances of anti-amyloid drugs, such as aducanumab and lecanemab were closely informed by the biomarker data showing target engagement and disease alteration. These approvals are a major advancement in AD treatment and make clear the role of biomarkers in the approval process (Thal et al., 2006).

### 8.2 Current trends in AD clinical trials

Investigations conducted over the last few years have observed the likelihood of biomarkers being used as endpoints in AD clinical trials. A review of 1,048 clinical trials revealed that around 30% utilized biomarkers as the first endpoint and around 35% as secondary (Hampel et al., 2017). The most used biomarkers involved amyloid-PET, tau-PET, and MRI. Phases of trials employing biomarkers were most representative in the first and second phase trials, where the biomarkers were used for the determination of safety and proof of concept. It has been evidenced from the current literature that biomarker usage in clinical trials is on the rise among researchers. In phase 2 trials, biomarkers can be an endpoint of the study, while in phase 3 trials would be used to support the primary study endpoint. It is a relatively new blood test called the NULISAseq CNS disease panel 120 that was developed at the University of Pittsburgh for diagnosing AD at the earliest stages, with only a blood sample and not utilizing any other biological fluids, with ability to detect over a hundred biomarkers simultaneously, providing a complete picture of what transpires within the AD of the brain (Blennow and Zetterberg, 2018).

### 8.3 Critical appraisal of biomarker-guided trials and translational challenges

While numerous recent clinical trials have underscored the potential of blood-based biomarkers in AD diagnosis and therapeutic monitoring, a balanced evaluation must consider negative or discrepant trial outcomes that highlight ongoing translational challenges. Several pivotal trials targeting amyloid clearance-such as verubecestat and solanezumab-demonstrated effective target engagement measured by amyloid PET or biomarker shifts but failed to show significant clinical benefit in cognition or disease progression (Egan et al., 2019; Doody et al., 2014). These findings raise critical questions about surrogate endpoint validity, timing of intervention, and patient selection.

Additionally, variability and lack of standardization in biomarker measurements-including plasma phosphorylated tau and Aβ ratios-pose challenges for reproducibility and clinical interpretation across diverse populations and study designs (Palmqvist et al., 2024; Pichet Binette et al., 2023). Discordances between cerebrospinal fluid, plasma, and imaging biomarkers in some trials further complicate reliance on single modalities. Lessons learned emphasize the need for earlier, preclinical-stage interventions; consensus on validated surrogate endpoints that reliably predict meaningful clinical outcomes; improved patient stratification addressing disease heterogeneity; and harmonization of biomarker assays and cut-off values. Embracing both successes and failures in trial outcomes is essential to refining biomarker-guided AD therapeutics and to accelerating their translation into clinical practice. The thorough details of clinical trials conducted by numerous researchers are explained in Table 3. This table provides a critical synthesis of pivotal clinical investigations assessing the diagnostic, prognostic, and disease-monitoring utility of BBBM in AD. Each study entry details the trial acronym or name, primary research objectives, principal findings regarding biomarker accuracy (e.g., sensitivity, specificity, predictive value), specific biomarkers analyzed (including phosphorylated tau isoforms [p-tau181, p-tau217], Aβ42/40 ratio, NFL), methodological framework (such as validation against cerebrospinal fluid or amyloid PET imaging, prospective real-world cohorts, or application of artificial intelligence-driven analytics), and translation into clinical or research practice. Collectively, these studies underscore the emerging reliability of minimally invasive blood biomarkers as diagnostic and prognostic tools, supporting their integration into routine clinical stratification, early detection, and individualized therapeutic approaches in AD.

**Table 3:** Key clinical studies evaluating blood-based biomarkers in alzheimer’s disease.

Study/Trial	Focus	Key findings	Biomarkers assessed	Methodology	Implications	References
NULISAseq CNS Disease Panel 120	Blood-based biomarker detection	Validated a new blood test capable of measuring over 100 biomarkers simultaneously, aiding early detection of AD.	Phosphorylated tau, amyloid beta, neuroinflammation markers, vascular health indicators	Analyzed 113 blood samples from cognitively normal adults are validated against classical AD biomarkers	Provides a less invasive method for detecting AD progression and potential for serial testing.	El-Sappagh et al. (2021)
ADBC Model	Personalized prediction of AD progression	Developed a model using real-world data to predict disease progression based on individual biomarker patterns.	CSF markers, imaging data, and memory tests	Utilized data from over 800 participants in the AD Neuroimaging Initiative (ADNI).	Enhances personalized treatment strategies by identifying unique patterns in biomarker changes over time.	El-Sappagh et al. (2021)
A/T/N Classification System	Biomarker classification for AD	Proposed a descriptive classification scheme categorizing biomarkers into three binary classes: A (amyloid), T (tau), and N (neurodegeneration).	Aβ (amyloid PET, CSF Aβ), p-Tau (CSF), neurodegeneration markers (FDG-PET, structural MRI)	Framework to categorize biomarker findings regardless of clinical diagnosis.	Facilitates understanding of complex biomarker profiles in AD research and clinical practice.	Ackley et al. (2024)
Classical Biomarkers Study	Diagnostic utility of classical biomarkers	Identifying key biomarkers (Aβ42, t-Tau, p-Tau) with significant diagnostic value for AD highlights differences in CSF vs. plasma levels.	Aβ42, t-Tau, p-Tau, NFL	Review of existing literature on biomarker effectiveness in diagnosing AD.	Supports the use of specific biomarkers for early diagnosis and monitoring of AD progression.	de Jong et al. (2007)
Blood Biomarkers in Clinical Practice	Clinical application of BBBM	High diagnostic accuracy for plasma p-tau assays in distinguishing AD from other neurodegenerative diseases is predictive of future dementia development.	Plasma p-tau levels, inflammatory markers	Analysis of various studies assessing BBBM in clinical settings.	Reducing reliance on invasive procedures like CSF sampling enhances early identification of pre-symptomatic AD.	de Jong et al. (2007); Ackley et al. (2024)

### 8.4 Novel prospects

The future of biomarker research in AD is promising, with several emerging key directions. But their standardization is essential for a set of biomarker measurement protocols for better comparison across studies and different centres. Longitudinal studies are subsequent studies that should employ prospective designs to assess changes in biomarker levels concerning cognition (Cedazo-Minguez and Winblad, 2010; Klyucherev et al., 2022). This approach enhances the causal relationships between biological determinants and disease progression. Diversity in clinical trials should be effectively maintained so that the results of clinical trials can be generalized for different populations. Integration with digital health technologies allows estimation of biomarker levels and cognitive status on an outpatient basis (Assfaw et al., 2024).

## 9 Conclusion

Unlike previous literature, this review uniquely integrates nutritional, inflammatory, and metabolic determinants to explain the variability of BBBM in AD. We map specific gaps and unresolved controversies, such as conflicting evidence on micronutrient interventions, lack of adjusted biomarker reference ranges, and poorly characterized phenotype-specific inflammatory profiles, to highlight that the simple cross-sectional biomarker values are insufficient for accurate, individualized AD diagnosis and risk stratification.

Our proposed model combines cross-domain synthesis, multi-omics integration, and AI-driven analytics to support precision medicine approaches for biomarker interpretation. By considering the interplay between nutrition, inflammation, and metabolism, this review establishes an actionable framework for future biomarker-guided clinical practice and research.

### 9.1 Summary of key findings

The levels of some biomarkers associated with AD are regulated by many interrelated processes that include diet, inflammation, and metabolism. Food determines the balance of inflammation and metabolism in the body, a factor that can determine if the person with AD will get worse or not. To overcome such limitations, researchers need to understand how certain diets may prevent age-related cognitive decline, including memory loss. It should be envisioned that integrating nutrient information into clinical practice may enable consumers in AD studies to adopt diets tailored towards the preservation of the brain and delay the progression to AD.

### 9.2 Future directions in biomarker research

The future trends in biomarker research associated with AD are well explained regarding the multi-omics approach, non-invasive techniques, the application of AI, and techniques related to inflammation and metabolism. These innovations help researchers go further in defining the biological factors of AD and develop better methods of early diagnosis, as well as a variety of specific targeted treatments. In the future, it may be important to develop ethical considerations and simultaneously make sure that everyone has equal opportunities to benefit from these technologies. The common goal is to develop a conceptual model that shapes the advanced understanding of AD while accounting for individual differences and that proposes interventions designed to reduce the identified risk factors of this heterogeneous condition.

## Glossary

AD: Alzheimer’s disease
AI: Artificial intelligence
CSF: Cerebrospinal fluid
BBB: Blood–brain barrier
BBBM: Blood-based biomarkers
MCI: Mild cognitive impairment
Vit. D: Vitamin D
Vit. E: Vitamin E
Vit. B: Vitamin B
ROS: Reactive oxygen species
T2DM: Type 2 diabetes mellitus
CRP: C-reactive protein
CNS: Central nervous system
Met-S: Metabolic syndrome
MCP-1: Monocyte chemotactic protein-1
MLA: Machine learning algorithms
IL: Interleukin
ADBC: Alzheimer’s disease biomarker cascade
PM: Personalized medicine
NFL: Neurofilament light
ADNI: Alzheimer disease neuroimaging initiative
APP: Amyloid precursor protein
BDNF: Brain-derived neurotrophic factor
TREM2: Triggering receptor expressed on myeloid cells 2
